# Babies, Their Food and Sleep

**Published:** 1872-10

**Authors:** 


### BABIES--THEIR FOOD AND SLEEP.

Give a baby plenty of food, of the proper quality; plenty of sleep, at regular intervals; plenty of flannel, of suitable length, and your baby should be healthy and jolly. Babies become as much addicted to habits as old folks, and if you pat them on their backs and sing lullabys in their ears, every time they roll over in the cradle, or give a little grunt indicative of restlessness, be sure they always will expect such services of you, and will demand it accordingly.

Most mothers or aunties have great times putting babies to sleep, and if you should be writing an article for a health magazine *down* stairs, you would imagine that a decidedly unhealthy one had exploded *up* stairs, were you to judge from the racket produced in soothing baby into his evening sleep. We believe, because we have seen it demonstrated again and again, that no such measure is necessary, but that babies can be taught habits quite as early as children of larger growth. They should be fed at regular hours, and not every time they wake or fret and cry. Babies cry quite as often from being over fed as from hunger,and many times the derangement of the bowels, colic, and other unpleasant symptoms common to infants, are directly attributable to the habit of cramming their little bellies with food, simply as a "soothing process," or for the reason that the mother does not know what else to do to produce peace and quietness in the household. Babies' stomachs are even more sensitive than grown people's, and can digest but a certain amount of food, while the surplus lodged there, from the stuffing process, must pass into the bowels in the form of undigested material, there to give rise to the various pains and aches which mothers are so familiar with. Therefore, use common sense in the feeding of your baby, and don't force food into its little stomach for every complaint it happens to make.

Baby should have its sleeping hours, which should be regulated according to its age. Quite young babies should sleep nearly *all* the while—that is their chief design. Those who have attained one year of age can be taught to have their sleeping and feeding hours, and will indicate them as regularly as your best time piece. Should the baby sleep all night, he should have a long nap in the middle of the day, and then kept awake until six or seven o'clock in the evening, when he should be placed in his crib and left to go to sleep without rocking,trotting, walking, swinging or other hallabaloo. He may rebel against such treatment a few times, but he will very soon learn that no attention will be paid to his outcries, and will fall to sleep in spite of himself. Let this plan be persisted in by mothers, before they have established bad habits in their babies; good ones are quite as easily obtained, when the mother and aunties will be surprised to find how little trouble a baby really is in the house when properly managed.

